# Research advances on postbiotics in promoting skin wound healing: mechanisms, formulations, and clinical translation

**DOI:** 10.3389/fimmu.2026.1912496

**Published:** 2026-08-27

**Authors:** Yu Xing, Jinyu Song, Bingbing Shang, Guangdi Yang, Ye Zhao, Ming Li, Binbin Hou

**Affiliations:** 1 Department of Dermatology, The Second Hospital of Dalian Medical University, Dalian, China; 2 Emergency Department, The Second Hospital of Dalian Medical University, Dalian, China; 3 College of Basic Medical Science, Dalian Medical University, Dalian, China

**Keywords:** fermented herbal medicines, immunity, microbiome, postbiotics, skin wound healing

## Abstract

Wound healing is a complex process involving different cell types, molecules and stages in the dermis, epidermis and associated microbiome. Chronic wounds caused by pressure, diabetes, or immunodeficiency often have huge economic and therapeutic costs. Postbiotics, in particular inactivated bacteria, cell components and metabolites, are emerging as novel treatments targeting the skin microbiome and provide precise treatment options for wound healing, inflammation, angiogenesis and re-epithelialization. We review the positive regulatory role of different microbial communities in skin healing; evaluate evidence and mechanisms of various postbiotics, *in vitro* and *in vivo* models as well as preliminary clinical studies; and summarize progress in the translation of postbiotics for skin wound repair.

## 1 Introduction

The human microbiome, consisting of various microorganisms across the body, is vital for health. The skin microbiota is crucial for skin balance and barrier function, with imbalances linked to skin disorders like dermatitis, psoriasis, and acne (1–3). Evidence indicates that the skin microbiome is also key in wound healing, involving interactions among epidermal cells, fibroblasts, vascular endothelial cells, and immune cells. Open wounds are readily colonized by microorganisms originating from the skin surface or the external environment. When the local microbial ecosystem becomes imbalanced, such colonization can predispose the wound to infection, delay healing, and even contribute to the formation of chronic wounds (4). Common pathogens, particularly Staphylococcus aureus and Pseudomonas aeruginosa, markedly impair wound repair through biofilm formation, virulence-factor secretion, quorum sensing, immune evasion, and sustained inflammatory activation (5–7).

Conversely, selected commensal microorganisms and probiotics can actively support wound repair by modulating immune responses, strengthening barrier function, and producing bioactive factors that promote tissue regeneration (8–10). Several pro-healing strains and their metabolites have therefore been explored as therapeutic candidates. Current bacteriotherapy strategies primarily rely on live microorganisms, including *Lactobacillus plantarum*, *Lactobacillus reuteri*, and *Saccharomyces boulardii*, all of which have shown beneficial effects on wound healing (11–13). Moreover, multi-strain formulations may further enhance repair by acting on key cellular mediators of healing, including neutrophils, macrophages, and fibroblasts (14, 15).

However, the clinical translation of live microbial therapeutics is constrained by safety concerns, limited stability, and demanding storage conditions. These limitations have driven growing interest in postbiotics, as a safer and more stable alternative for wound management. According to the International Scientific Association of Probiotics and Prebiotics (ISAPP) consensus definition, postbiotics are “preparations of inanimate microorganisms and/or their components that confer a health benefit on the host”. They include inactivated bacteria, cellular components, lysates, and microbial metabolites, but do not contain viable microorganisms as the active entity (16, 17). Emerging evidence indicates that specific postbiotics can promote wound repair. Particularly noteworthy are postbiotics generated through probiotic fermentation of herbal ingredients, which may enhance the bioactivity of herbal substrates. For instance, fermentation has been shown to increase the antioxidant activity of Salvia miltiorrhiza and chamomile, while extracts from the fermented substrate of *Isaria cicadae* promote hair follicle regeneration via modulation of the Hippo signaling pathway (18, 19).

In this review, we focus on the dynamic alterations of the skin microbiota during skin wound healing, summarize the mechanisms by which different postbiotic formulations promote wound repair, and discuss the major challenges facing their clinical translation. By doing so, we aim to provide a theoretical foundation and practical reference for the development of novel wound-healing strategies.

## 2 Wound healing process and treatment strategies

### 2.1 Fundamental processes of wound healing

Wound healing is a highly coordinated, multistage physiological process, including blood clot formation, inflammatory cell infiltration, fibroblast migration, epidermal re-epithelialization, and tissue remodeling. Clinically, it is generally divided into three interrelated and partially overlapping phases: the inflammatory phase, the proliferative phase, and the remodeling phase (20).

After skin injury, platelets form a provisional clot, followed by the recruitment of inflammatory and reparative cells, including neutrophils, macrophages, stem cells, fibroblasts, keratinocytes, and endothelial cells, which jointly drive inflammation, proliferation, and tissue reconstruction (21–23).

The proliferative phase is a critical stage of wound healing and is characterized by granulation tissue formation, angiogenesis, re-epithelialization, and wound contraction. Fibroblasts generate granulation tissue and coordinate these processes by synthesizing and depositing the extracellular matrix (ECM) to support cell migration and proliferation, while activating signaling pathways such as FAK–ERK–MCP1, Wnt, transforming growth factor-beta (TGF-β), platelet-derived growth factor (PDGF), and resistin-like molecule alpha (RELMα), along with cytokines and growth factors that drive cell activation and tissue remodeling (24–27). Concurrently, neovascularization, keratinocyte and epidermal stem cell-mediated epidermal repair, and myofibroblast-dependent contraction contribute to vascularization, re-epithelialization, and wound contraction, respectively (28–31).

Based on healing duration, wounds are classified as acute or chronic. Acute wounds generally heal within 4–12 weeks, whereas chronic wounds are associated with impaired growth factor secretion, excessive reactive oxygen species (ROS) and matrix metalloproteinase (MMP) production, reduced cellular mitotic activity, delayed healing, infection risk, and abnormal scar formation (32).

### 2.2 Clinical treatment strategies for wound healing

Clinical wound-healing therapies promote tissue repair by modulating the wound microenvironment and activating pro-regenerative signaling pathways (33). Among these approaches, hydrocolloid, foam, and hydrogel dressings support angiogenesis, fibroblast activation, and extracellular matrix deposition mainly through HIF-1α/VEGF, PI3K-Akt, MAPK/ERK, and TGF-β/Smad signaling (34, 35). Silver-containing dressings provide antimicrobial activity while regulating inflammation and oxidative stress via NF-κB and Nrf2 pathways (36, 37).

In parallel, negative pressure wound therapy enhances fibroblast migration, collagen remodeling, and angiogenesis through FAK, Rho GTPase, YAP/TAZ, and HIF-1α related pathways (38–40). Growth factor therapies, such as rhEGF and rhPDGF, activate RTK-mediated Ras/Raf/MEK/ERK and PI3K-Akt signaling pathways, thereby accelerating granulation tissue formation and re-epithelialization (41, 42). Biological scaffolds promote tissue regeneration through integrin-YAP/TAZ signaling (43).

Although current therapies target key repair-related pathways and provide new options for wound management, their side effects and high costs limit broader clinical application, highlighting the need for safer and more cost-effective strategies. The advantages and limitations of these therapies are summarized in **Table 1**.

**Table 1:** Advantages and disadvantages of modern wound healing therapies.

Modern clinical therapy	Advantages	Disadvantages	Reference
Hydrocolloid/Foam Dressings	- Maintain moist environment - Absorb low to moderate exudate - Barrier against contamination - Easy to use and cut to fit	- Not for heavily exuding or infected wounds - May damage new epithelium upon removal - Limited effectiveness for deep or tunneled wounds	(34, 44–46)
Hydrogels	- Hydrate dry wounds, promote autolytic debridement - Cooling, pain-relieving effect - Suitable for dry or low-exudate wounds	- Do not absorb exudate - Require secondary dressing - Not suitable for highly exuding wounds	(35, 47)
Silver-containing dressings release silver ions (Ag^+^)	- Broad-spectrum antimicrobial (Ag^+^ release) - Reduce infection risk, especially in colonized wounds	- Potential cytotoxicity with prolonged use - Higher cost - May stain skin or clothing	(36, 37, 48)
Negative Pressure Wound Therapy (NPWT)	- Promotes granulation, removes exudate - Reduces edema, improves perfusion - Aids wound contraction	- High equipment and supply costs - Requires trained application and monitoring - Contraindicated in untreated necrotic tissue, active bleeding, or malignancy	(38–40, 49)
Exogenous growth factor therapies	- Directly stimulate cell proliferation and angiogenesis - Useful for chronic, hard-to-heal wounds	- High cost, limited stability - Requires appropriate wound bed preparation - May be degraded by proteases in the wound	(41, 42)
Biological scaffolds	- Provide 3D structure for cell migration and ingrowth - Can deliver bioactive molecules	- Potential immunogenicity (xeno/allo-grafts) - Degradation rate must match tissue regeneration - Can be costly and complex to produce	(43)

## 3 Role and mechanisms of the skin microbiome in wound healing

### 3.1 Characteristics of the skin microbiome

The surface of human skin is colonized by a large number of microorganisms, including bacteria, fungi, Archaea, viruses and mites, etc (50). According to the colonization characteristics, they can be divided into resident symbiotic bacteria and transient bacteria. The distribution of skin microorganisms is site-specific and is affected by environmental factors such as temperature, humidity, and pH. For example, the detection amounts of *Staphylococcus epidermidis*, *Cutibacterium acnes*, *Staphylococcus aureus*, *Corynebacterium*, and fungi in epidermal specimens are more than those in full-thickness skin specimens. In addition, people of different ethnicities, genders, and ages also show different characteristics of skin microbiota distribution. Some studies have found that with age, the number of S. *epidermidis* decreases while the number of S. *aureus* increases (51).

Generally, the amounts of S. *epidermidis*, Corynebacterium, and C. *acnes* in men are higher than those in women, while the contents of S. *aureus* and fungi are lower than those in women (52, 53). The resident microbiota of healthy skin maintains the microecological balance by resisting foreign pathogens and regulating immune responses.

### 3.2 The role of normal skin microbiota in maintaining skin health

The normal skin microbiota, a complex community of microorganisms residing on the skin surface, plays several essential roles in maintaining skin health and homeostasis (54).

Barrier Function and Microbial Defense

Pathogen Competition: Resident microbes inhibit pathogenic colonization (e.g., by S. *aureus*) through niche occupation, nutrient competition, and production of antimicrobial peptides (AMPs) (55); Chemical Barrier: Microbial metabolites (e.g., short-chain fatty acids, lactic acid help maintain the skin’s acidic pH about 5.5, which suppresses pathogen growth (56, 57).

Immune Regulation and Homeostasis

Immune Training: The microbiota educates and modulates the local immune system, promoting tolerance and preventing excessive inflammation (58); Anti-inflammatory Effects: Certain species (e.g., S. *epidermidis*) can induce regulatory T cells and anti-inflammatory cytokines (e.g., IL-10), maintaining immune balance.

Promotion of Wound Healing

Beneficial microbes and their byproducts can enhance re-epithelialization, modulate inflammation, and promote angiogenesis by activating immune response pathways, thereby optimizing the wound healing process (8, 56).

Maintenance of Microbial Balance

Although many dermatological conditions, such as atopic dermatitis and acne, are associated with disruptions in skin microbiota homeostasis, a high degree of microbial diversity and a balanced community structure confer resilience to the ecosystem. This resilience enhances its ability to withstand disturbances, such as antibiotics and harsh cleansers, and to restore homeostasis, thereby reducing the risk of disease development. This mechanism holds significant importance for maintaining skin health (59, 60).

### 3.3 Dual regulation of wound healing by the skin microbiome

#### 3.3.1 Inhibitory effects of the specific skin microbiota on wound healing

The immunomodulatory effect of microorganisms during the wound healing process is stage-specific. In the early stage of inflammation, microorganisms interact with pattern recognition receptors (PRRs) on the surface of immune cells through their pathogen-associated molecular patterns (PAMPs), exerting a negative regulatory effect (61, 62). When the microbial load is moderate, the inflammatory response can be effectively initiated and optimized to promote the debridement function. However, when a biofilm is formed or microorganisms overproliferate, the continuously released PAMPs lead to the overactivation of the innate immune signaling pathway, blocking the polarization shift of macrophages from the pro-inflammatory M1 type to the anti-inflammatory and reparative M2 type. This keeps macrophages in a pro-inflammatory state, resulting in the massive release of inflammatory factors such as IL-1β and TNF-α and the recruitment of excessive neutrophils (63–66). This transforms acute inflammation into a chronic and destructive pathological state (67).

After entering the proliferation phase, microorganisms impede the repair process through multiple mechanisms. Biofilms form a physical barrier that hinders the migration of fibroblasts, keratinocytes, and endothelial cells towards the wound center, obstructing re-epithelialization and granulation tissue formation, while evading the recognition and clearance of the body’s immune system (67–69). Some bacterial biofilms inhibit microRNA-143 (miR-143) and upregulate MMP-2, leading to the degradation of type I collagen and thus disrupting the structure and function of granulation tissue (70). Microorganisms competitively consume oxygen and nutrients, causing local hypoxia and acidosis (67, 71). Even after the wound is closed, the previous excessive inflammation and abnormal repair can still result in disordered fibroblast arrangement and significantly increase the risk of scarring (72, 73). The latent microorganisms within the biofilm may also become re-activated, leading to repeated wound breakdown (74).

Recent work shows that control of the equilibrium of the whole community is crucial for maintaining local immune homeostasis via competitive inhibition and immunomodulation (75–77), while dysbiosis (a slow diversity of bacteria and a majority of pathogenic species, such as S. *aureus* and *Pseudomonas aeruginosa*) leads to pathological immune responses, which escape phagocytosis and provoke aberrant Th17 cells and chronic wounds.

#### 3.3.2 Promotive effects of specific skin microbiota on wound healing

In recent years, studies have found that under specific conditions, certain pathogenic bacteria or their specific components can indirectly promote wound healing, reflecting the complexity of host-microorganism interactions.

Some microorganisms in the normal skin microbiota have an antagonistic effect on pathogenic bacteria, thereby achieving an anti-inflammatory effect. For example, certain Gram-positive bacteria can produce antibacterial substances such as bacteriocins to inhibit the growth of pathogenic bacteria like S. *aureus* and *Escherichia coli* (78). These resident microbiota occupy ecological niches, form biofilms to antagonize pathogenic bacteria, protect the wound surface, reduce the risk of pathogenic bacteria infection, and promote normal wound healing. As early as the end of the last century, the ecological preparation (skin probiotics) developed by the research team led by Professor Xiong Dexin in China was found in *in-vitro* and *in-vivo* experiments to have the ability to antagonize P. *aeruginosa*, S. *aureus*, and E. *coli* on the burn wounds of mice, reduce the wound infection rate, and shorten the healing time. Compared with silver-zinc cream, a commonly used burn medicine, this ecological cream has a clear curative effect, long-lasting action, no sensitization, and high safety. In addition, research has found that the fermentation supernatant of *Micrococcus luteus* YM-4 derived from human skin can up-regulate Claudin 1 (CLDN1), Filaggrin (FLG), and Aquaporin 3 (AQP3), strengthen the skin barrier and skin hydration, and reduce water loss. It can also up - regulate Hyaluronan Synthase 3 (HAS3) to increase hyaluronic acid synthesis, maintaining the structure and humidity of ECM. Under stress conditions, it also reduces the inflammatory markers (such as IL-1β, IL -6, TSLP, COX2, S100A19) of keratinocytes and fibroblasts, alleviates UVB-induced damage, reduces collagen degradation by inhibiting the expression of MMP-1, and restores the expression of Type I Collagen (COL1A1), promoting collagen synthesis (79).

With the in-depth research, it has been found that although the presence of pathogenic bacteria can induce wound inflammation, the complete absence of these bacteria (such as in a sterile state) can instead impede the wound-healing process. This indicates that the commensal microbiota is an important factor in immune regulation and the promotion of wound healing (80–82). When the epidermis is damaged, the surrounding commensal microbiota will colonize the dermis of the injured skin, activating neutrophils to express the chemokine CXCL10 and recruiting plasmacytoid dendritic cells (pDC). The pDC activates Toll-like receptor 9 (TLR9) by binding to bacterial DNA, producing a large amount of type I interferons, which stimulate fibroblasts and macrophages to produce growth factors to promote wound healing (83). S. *aureus* can stimulate M2 macrophages and CD4^+^ T cells through the inflammatory cytokine IL-1β and the keratinocyte-dependent IL-1R-Myeloid differentiation factor 88 (MyD88) pathway, inducing tissue regeneration (84). Studies have also found that its quorum-sensing system, the Accessory gene regulator (Agr), and the autoinducing peptide (AIP) it secretes can activate specific receptors on keratinocytes, prompting keratinocytes to produce AMPs and cytokines, thus enhancing the skin’s defense ability.

Flagellin of P. *aeruginosa* can trigger inflammation by activating TLR5 (74), promote the proliferation and migration of keratinocytes, and stimulate the expression of AMPs, thereby accelerating wound healing and enhancing resistance to other pathogens (85, 86).

S. *epidermidis* can “train” resident skin T cells and keratinocytes, enabling them to generate a more rapid and effective immune response when encountering injuries (87). Further research has found that S. *epidermidis* can induce CD8+ T cells to regulate specific immune responses, upregulate the expression of genes related to tissue repair, and promote wound healing; its lipoteichoic acid can act on TLR3, which helps regulate excessive inflammation (8). It can also induce a protective interferon response through its unique phenol-soluble modulin γ (PSMγ), activate the interferon (especially type I interferon) signaling pathway in host cells, and promote the migration and proliferation of keratinocytes, thereby accelerating wound repair. Some strains of S. *epidermidis* can also produce lantibiotics, which specifically inhibit the growth of S. *aureus* and create a “clean” environment for wounds (81). In addition, Gram-negative bacteria, such as *Alcaligenes faecalis*, can enhance the activity of keratinocytes by producing angiogenesis-promoting factors and promoting the inflammatory cytokine IL-8, thus promoting wound healing (88).

**Figure 1:**
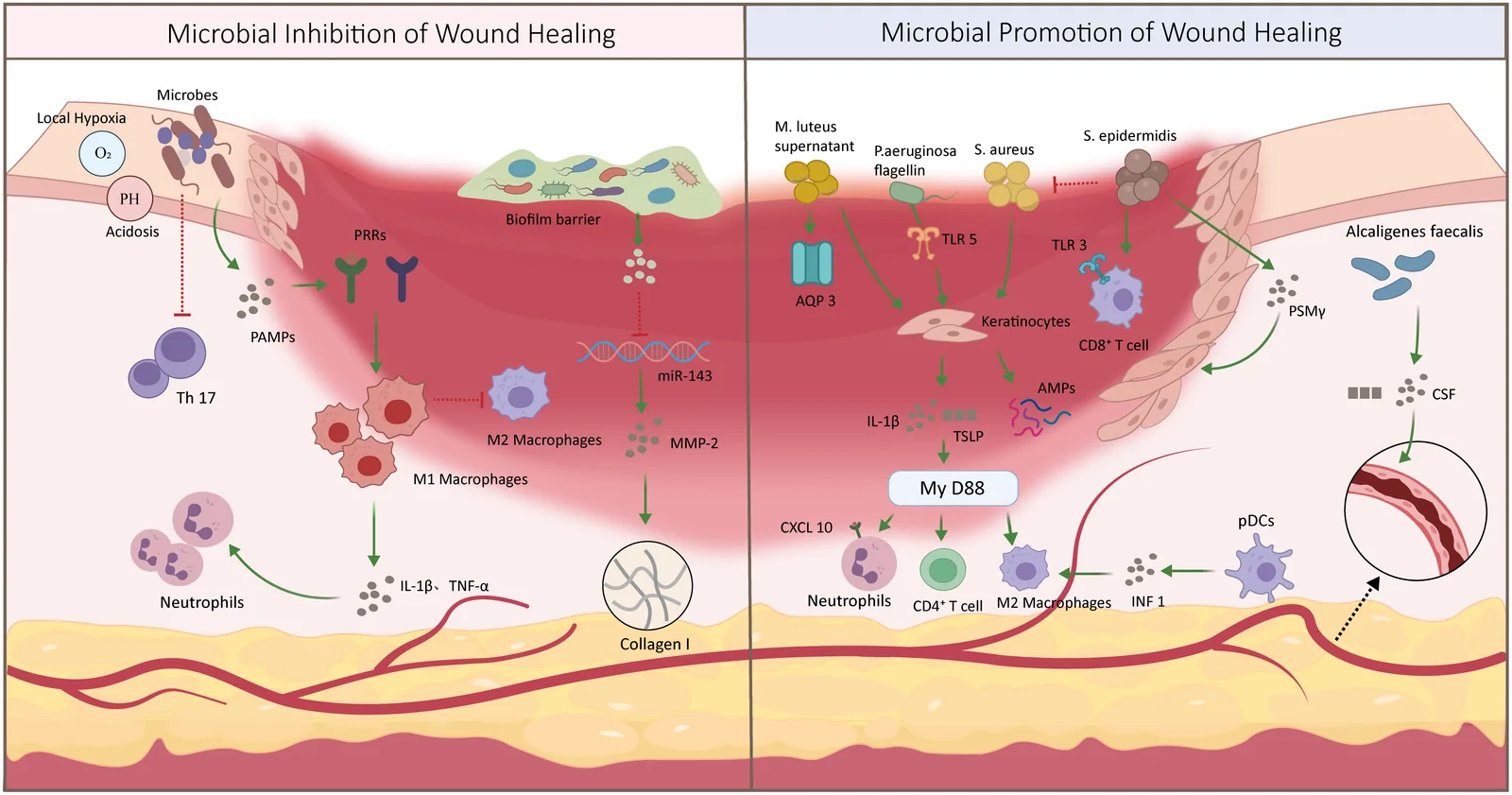
Schematic of the dual regulation of wound healing by the skin microbiome.

These mechanisms uncover the extremely complex symbiotic, antagonistic, and utilization relationships between microorganisms and the host, offering new perspectives for the development of novel therapeutic strategies based on attenuated bacterial cells, specific bacterial components, or quorum sensing regulation. **Figure 1** depicts the dual effects of the skin microbiome during the wound healing process.

#### 3.3.3 The role of the skin microbiota in inflammatory skin disorders

The skin microbiota functions not merely as a regulator of infectious wound repair, but as a central determinant of chronic inflammatory skin disorders, including atopic dermatitis (AD), hidradenitis suppurativa (HS), and psoriasis. Beyond local dysbiosis, these conditions involve intricate host–microbe crosstalk; notably, the gut–skin axis reveals a bidirectional regulatory network wherein perturbations in either compartment drive cutaneous pathology (89, 90).

In HS, follicular occlusion in intertriginous sites creates a hypoxic niche favoring *Corynebacterium* and obligate anaerobes, triggering neutrophilic inflammation and sinus tract formation—a vicious cycle exacerbated by endocrine and metabolic stressors (91). Similarly, acne pathogenesis extends beyond C. *acnes* overgrowth to a meta-inflammatory paradigm integrating hyperkeratinization, dyslipidemia, and hormonal dysregulation, necessitating metabolic interventions (92, 93). Psoriasis exemplifies immune–microbial convergence, where IL-23/IL-17 axis activation is amplified by S. *aureus* colonization and gut dysbiosis, driving keratinocyte hyperproliferation via Th17 differentiation (94, 95).

Thus, the skin microbiota has evolved from a passive bystander to a master regulator of cutaneous immune homeostasis. Its dysregulation underpins both reparative and pathological processes, while the gut–skin axis provides a conceptual framework for systemic therapeutic targeting beyond topical interventions.

**Figure 1** illustrates the physiological activities of the skin surface microbiome during wound formation. It highlights the dual role of skin pathogenic microbiota in wound healing. On one hand, when biofilms or microorganisms proliferate excessively, PAMPs become overactivated, placing macrophages in a persistent pro-inflammatory state that leads to chronic inflammation. Simultaneously, biofilms act as physical barriers, suppressing the activity of wound-associated cells and thereby impeding wound healing. On the other hand, the skin pathogenic microbiota can promote wound healing to some extent by regulating biofilm formation, community effects, immune activity, and specific bacterial proteins such as flagellin and phenol-soluble modulin γ (PSMγ). However, bacteriocins produced by the commensal microbiota and their acellular supernatants can exert antibacterial effects, thereby achieving the same promotional outcome.

## 4 The potential of postbiotics in wound healing

The impairment of wound healing resulting from skin microbiota dysbiosis underscores the critical role of microbial communities in maintaining skin homeostasis. In recent years, growing research attention has focused on whether microbial-based preparations can directly or indirectly modulate the skin microbiota to influence wound healing progression. Within this research direction, investigations into postbiotic preparations have gained particular prominence.

### 4.1 Definition of postbiotics

Postbiotics have gained prominence as a crucial concept in microbiome-based interventions. In 2021, ISAPP formally defined postbiotics as “a preparation of inanimate microorganisms and/or their components that confers a health benefit on the host” (96). This definition underscores two fundamental criteria: firstly, postbiotics must originate from inactivated microorganisms and/or their associated components; secondly, their health benefits must be substantiated by experimental or clinical evidence. In contrast to earlier, more expansive interpretations—which often included fermented metabolites, cell-free supernatants, or merely inactivated microbes—the ISAPP consensus offers a more precise conceptual framework and establishes more stringent scientific parameters for the term “postbiotic” (16, 96).

### 4.2 Composition and classification of postbiotics

According to current consensus and available evidence, postbiotics are not single entities but rather complex preparations composed of multiple bioactive substances derived from inactivated microorganisms (97, 98). Their principal forms include inactivated whole cells, such as lactic acid bacteria or mycobacteria rendered non-viable by heat, high pressure, irradiation, or ultrasound; structural components of microbial cells, including teichoic acids, peptidoglycan, lipoteichoic acid, surface proteins, capsular polysaccharides, cell wall fragments, and exopolysaccharides; and bioactive metabolites generated during fermentation, such as organic acids, short-chain fatty acids, antimicrobial peptides, vitamins, and amino acids (99).

Notably, the concept of postbiotics has expanded from products derived solely from classical probiotics to functional preparations generated by microbial fermentation of natural medicinal and food materials. In this context, fermented herbal medicines represent an important extension of postbiotic research. These preparations contain not only inactivated or residual microbial components, but also bioactive metabolites produced through microbial biotransformation of herbal medicines, including small molecules, oligosaccharides, phenolic acid derivatives, and flavonoid aglycones (100). Such fermentation can markedly modify the solubility, bioavailability, and immunomodulatory properties of herbal constituents, yielding a composite bioactive system with both microbe-derived and herb-derived transformed components. This system may confer advantages in anti-inflammatory, antioxidant, antimicrobial, and tissue regenerative effects (101–103). Therefore, fermented herbal medicines may also fall within the scope of postbiotics.

### 4.3 Advantages of postbiotics

The best example of postbiotic application is an experiment on obese and overweight people, which first demonstrated that inactivated bacteria are more effective than live bacteria (104). Meanwhile, compared with live bacterial preparations, postbiotics have advantages such as higher safety, a wider range of applicable populations, and better stability (96, 105). Postbiotics do not contain live bacteria, so there is no risk of live bacteria colonization or infection, making them particularly suitable for patients with low immunity (106). Additionally, postbiotics are generally more stable and easier to store and transport.

In recent years, the role of the gut-skin axis in skin repair has received increasing attention. This axis indirectly regulates skin homeostasis and injury repair by integrating immune, metabolic, microbial, and neuroendocrine signals (11, 107, 108). The gut microbiota is closely associated with epithelial barrier integrity, nutrient metabolism, immune system maturation, and systemic inflammatory status. Oral administration of postbiotics may attenuate systemic inflammation by remodeling the gut microbial ecosystem and promoting the production of beneficial metabolites, such as short-chain fatty acids (SCFAs) (109). In addition, postbiotics can modulate adaptive immune responses, as reflected by the suppression of Th17 cell-associated inflammatory pathways and the promotion of regulatory T-cell differentiation, thereby restoring immune microenvironmental homeostasis (110). Previous studies have also shown that postbiotic intervention can reduce serum IgE and IFN-γ levels and inhibit mast cell infiltration (111). These findings suggest that the immunomodulatory effects of postbiotics are not confined to the intestinal compartment but may further influence systemic immune status by maintaining intestinal immune homeostasis, thereby indirectly contributing to skin homeostasis and tissue repair (89, 112).

However, oral postbiotics must undergo gastrointestinal processing, systemic circulation, tissue distribution, and immune or metabolic modulation before indirectly affecting the wound microenvironment, resulting in a relatively long route of action, limited local targeting, and delayed therapeutic onset. From a clinical perspective, topical intervention may overcome these limitations by acting rapidly at the wound site, establishing a local pro-repair microenvironment, and efficiently promoting tissue repair (113). Moreover, current studies on oral postbiotics have mainly focused on inflammatory skin diseases, such as atopic dermatitis, acne, and psoriasis, whereas direct evidence supporting their role in cutaneous wound healing remains limited (109, 114, 115). Therefore, topical postbiotics may offer greater application advantages and clinical translational potential than oral formulations for wound-healing therapy.

## 5 Research trends on postbiotics promoting wound healing

### 5.1 Postbiotics from lactic acid bacteria promote healing through multiple mechanisms

Lactic acid bacteria (LAB) are Gram-positive, non-sporing, catalase-negative organisms that typically appear as cocci or rods (116). They are fastidious, acid-tolerant, and primarily produce lactic acid from glucose. Many LAB also generate antimicrobial substances such as bacteriocins, hydrogen peroxide, and diacyl compounds. LAB genera including *Lactobacillus*, *Lactococcus*, *Leuconostoc*, *Pediococcus*, *Streptococcus*, *Aerococcus*, *Alloiococcus*, *Carnobacterium*, *Dolosigranulum*, *Enterococcus*, *Oenococcus*, *Tetragenococcus*, *Vagococcus*, and *Weissella* (117).

**Figure 2:**
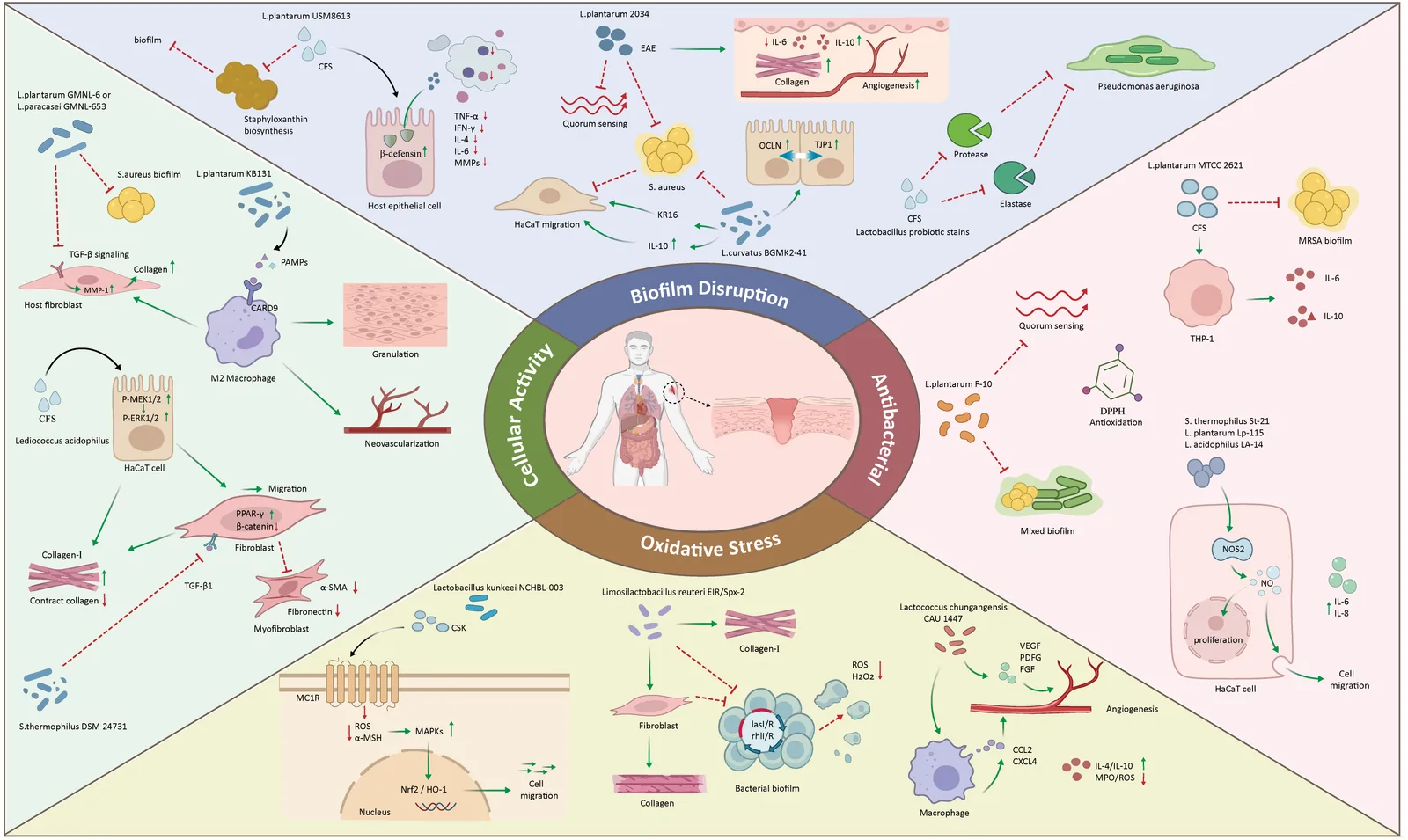
Schematic diagram of postbiotics derived from lactic acid bacteria regulating wound healing.

Various postbiotic components derived from LAB demonstrate a range of effects in facilitating skin wound healing, with the postbiotics of *Lactobacillus plantarum*, *Lacticaseibacillus casei*, and *Lacticaseibacillus rhamnosus* exhibiting notably significant impacts and playing a crucial role in skin regeneration (78, 118, 119). **Figure 2** shows the process by which different types of lactic acid bacteria postbiotics influence wound healing through multiple mechanisms.

#### 5.1.1 Regulating the activity of wound cells

W. H. Tsai et al. demonstrated that formulations containing heat-inactivated strains of *Lactobacillus plantarum* GMNL-6 or *Lactobacillus paracasei* GMNL-653 facilitate collagen synthesis by engaging the TGF-β/pSmad signaling pathways. This mechanism suppresses the expression of αSMA, Smad2/3, and phosphorylated Smad2, while enhancing the expression of MMP-1, thus promoting re-epithelialization and preventing the excessive accumulation of myofibroblasts (120). Additionally, heat-killed *Lactobacillus plantarum* KB131 facilitates chronic wound healing by driving CARD9-mediated M2 macrophage polarization, which subsequently promotes wound closure, re-epithelialization, and granulation tissue formation, while enhancing the accumulation of α-SMA secreted by myofibroblasts (121).

The cell-free supernatant (CFS-LS) derived from *Lediococcus acidophilus* has been shown to significantly enhance the phosphorylation levels of p-MEK1/2 and p-ERK1/2. Through the activation of the ERK pathway, it facilitates keratinocyte migration and increases collagen I synthesis (122). Research by F. Lombardi et al. demonstrated that lysates from *Streptococcus thermophilus* St-21, *Lactobacillus plantarum* Lp-115, and *Lactobacillus* LA-14 affect the keratinocyte (HaCaT) scratch model via mechanisms mediated by nitric oxide synthase 2. These lysates activates the NOS2/NO signaling cascade in HaCaT cell, thereby enhancing their proliferation and migration to accelerate re-epithelialization. Concurrently, this bacterial stimulus drives the upregulation of pro-inflammatory mediators, specifically IL-6 and IL-8 (123). Subsequently, F. Lombardi’s team conducted further research, extracting bacterial lysate from *Streptococcus thermophilus* DSM 24731. This lysate modulated the collagen contraction capacity in activated dermal fibroblasts by reducing the expression levels of α-SMA, fibronectin, and collagen I, while targeting TGF-β_1_ signaling. Additionally, by upregulating PPARγ, it decreased Smad2/3 activation, TGF-β_1_ mRNA levels, and β-catenin expression. This multi-targeted intervention in TGF-β_1_-mediated fibrotic signaling networks significantly inhibited skin fibrosis and reduced scar formation (124).

#### 5.1.2 Inhibition of oxidative stress

*Lactobacillus kunkeei* NCHBL-003 culture supernatant (CSK) activates the MAPK-Nrf2-HO-1 signaling pathway and promotes the scavenging of excessive ROS. Attenuation of ROS downregulates α-MSH expression via MC1R modulation. Concomitantly, this redox shift activates the MAPK signaling cascade, which in turn triggers the Nrf2/HO-1 pathway, ultimately enhancing cellular migratory capacity (125). The exopolysaccharide Eps extracted from *Lactobacillus plantarum* NIMBB003 can reduce oxidative stress, regulate the inflammatory response, and bind to the carbohydrate recognition domain receptors on the cell membrane, enhancing cell adhesion, spreading, and proliferation (fibroblasts) (126).

A recent study revealed that the postbiotics derived from *Limosilactobacillus reuteri* EIR/Spx-2, isolated from the gut microbiota of long-lived blind mole rats, can facilitate fibroblast activation and COL1A1 production. Concurrently, it suppresses the lasI/R and rhlI/R systems, thereby disrupting biofilm formation and quorum sensing. Furthermore, it markedly attenuates ROS and H_2_O_2_ levels, establishing a potent antioxidant defense, while concomitantly downregulating TNF-α and IL-6 to resolve inflammation (127). Heat-killed *Lactococcus chungangensis* CAU 1447 reduces the activity of myeloperoxidase (MPO), decreases neutrophil-mediated oxidative stress, alleviates the inflammatory response, upregulates anti-inflammatory factors (such as IL-4 and IL-10), and regulates pro-inflammatory factors (such as TNF-α and IL-6) to regulate the inflammatory response. It also upregulates the expression of growth factors: VEGF, FGF, PDGF, and TGF-β1 to promote tissue regeneration and angiogenesis. At the same time, it regulates chemokines CCL2 (MCP-1) and CXCL4 (PF4) to attract repair-related cells such as monocytes and macrophages to migrate into the wound, accelerate the removal of necrotic tissue, and promote repair (128).

#### 5.1.3 Antimicrobial and biofilm-disrupting effects

Further research has found that postbiotics of Lactobacillus plantarum also interferes with the growth of Pseudomonas aeruginosa and Staphylococcus aureus. Ong JS et al. discovered that the protein-rich fermentation supernatant of *Lactobacillus plantarum* USM8613 can stimulate the expression of β-defensin at the wound site, promote innate immunity. And the coordinated suppression of pro-inflammatory cytokines (TNF-α, IFN-γ, IL-6) and MMPs, coupled with the reduction of the Th2 mediator IL-4, created a permissive microenvironment that facilitated inflammation resolution and promoted re-epithelialization. in wound healing (78). Some scholars isolated the supernatant of lactobacilli from infant feces and found that it had a significant inhibitory effect on Pseudomonas aeruginosa. The cell-free lysate (postbiotic) of *Latilactobacillus curvatus* BGMK2–41 exerts its pro-healing effects via two principal mechanisms: First, inhibition of S. *aureus* and KRI6 activation synergistically enhanced keratinocyte migration, thereby accelerating re-epithelialization. Second, upregulation of IL-10 facilitated cellular motility, while concurrently increasing Occludin (OCLN) and Tight Junction Protein 1 (TJP1) expression to restore the epidermal barrier (129).

Previous studies have shown that the cell-free extract of *Lactobacillus plantarum* F-10 inhibits the expression of soluble virulence factors, disrupts biofilm formation, and interferes with the quorum sensing of pathogens (130). Many researchers have investigated the antibacterial properties of MRSA. Narang et al. found that the ethyl acetate extract (EAE), a postbiotic derived from *Lactiplantibacillus plantarum* 2034, inhibits the growth of MRSA, disrupts established biofilms, interferes with quorum sensing, modulates inflammation by reducing the expression of the pro-inflammatory cytokine IL-6 and increasing the anti-inflammatory cytokine IL-10, accelerates tissue repair by promoting re-epithelialization, collagen deposition and angiogenesis, and decreases scar formation by decreasing the expression of TGF-1 (131). Dubey et al. reported that the cell-free supernatant of *Lactiplantibacillus plantarum* MTCC 2621 inhibits dose-dependent biofilm inhibition and anti-MRSA activity. *In vivo* studies with mice with MRSA-infected incision wounds showed reduction in IL-6 expression and increase of IL-10 expression (132).

### 5.2 Postbiotics of *Bacillus subtilis* promotes wound repair

Bacillus species and their components are exhibited to improve wound healing. For example, lipopeptide biosurfactant created by the *Bacillus subtilis* SPB1 promotes wound healing by organizing collagen fibers and fibroblasts (133). Serine proteases produced by *Bacillus subtilis* D10 clears necrotic barriers and re-establishes hemodynamics, thereby fostering neovascularization. Consequently, the recruitment of fibroblasts and keratinocytes drives robust collagen maturation and epidermal barrier restoration (134). Various studies have also reported that postbiotics made from *Bacillus subtilis* sp. *natto* ATCC 15245, prepared by various methods, have a positive effect on wound healing. In 2020, Y. Ashoori et al. demonstrated that a chitosan nanogel made from the lysates of *Bacillus subtilis* sp. *natto* ATCC 15245 has many bioactive metabolites (polysaccharides, organic acids), which promote matrix remodeling via proteoglycan synthesis and angiogenesis, while suppressing inflammation and potentiating growth factor (EGF/PDGF/FGF/TGF-β) signaling (135). In 2021, N. Golkar et al. found that a non-viable microbial bio-cold cream made from *Bacillus subtilis* sp. *natto* ATCC 15245 is effective in wound healing and has high hydroxyproline content (136).

**Table 2** summarizes the action of probiotic-derived postbiotics in wound healing. Studies have shown that even though probiotics differ in species, they may act similarly to promote the same stage of wound healing. Likewise, even postbiotics from the same source may have different regulatory effects at different stages and pathways. Combining postbiotics from different sources may represent a new strategy for wound healing.

**Table 2:** Multifaceted and stage-dependent mechanisms of microbial postbiotics in wound healing.

Bacterial producer	Preparation type	Experiment type	Skin wound model	Mechanism	References
Lactic acid bacteria Regulating the activity of wound cells					
*Lactobacillus plantarum* GMNL-6/Lactobacillus paracasei GMNL-653	Heat-killed	*In Vivo*、*In Vitro*	Wound healing mice model/Hs68 cells	Increased levels of Collagen、Remodeling	(120)
*Lactobacillus plantarum* KB131	Heat-killed	*In Vivo*	Wound healing mice model	Re-epithelialization、Granulation、Neovascularization	(121)
*Lediococcus acidophilus*	CFS-LS	*In Vitro*	B16F10 cells、 HaCaT cells	Increased levels of Collagen–I、HaCaT cells migration by P-MEK1/2,P-ERK1/2	(122)
*Streptococcus thermophilus* DSM 24731	Lysate	*In Vitro*	NHDF cells	Increased levels of collagen、Inhibition of the transformation from fibroblast to myofibroblasts by TGF-β1 signaling pathway	(124)
*Streptococcus thermophilus* St-21、 *Lactobacillus plantarum* Lp-115、*Lactobacillus* LA-14	Lysate	*In Vitro*	HaCaT cells	Re-epithelialization、Increased levels of inflammatory Factors(IL-6、IL-8)by NOS2/NO signaling pathway	(123)
Antibacterial and biofilm-disrupting					
*Lactobacillus plantarum* USM8613	CFS(Cell-free supernatant)	*In Vivo*	Wound healing mice model	Anti-Staphyloxanthin biosynthesis biofilm effect、Increased levels of β-defensin、Re-epithelialization	(78)
*Lactobacillus curvatus* BGMK2-41	Lysate	*In Vivo*、*In Vitro*	HaCaT cells/Human skin wound healing model	Anti-Staphylococcus aureus effect、Epidermal barrierincreased、Re-epithelialization	(129)
*Lactobacillus plantarum* F-10	CFE	*In Vitro*		Anti-Pseudomonas aeruginosa and MRSA biofilm effect、Anti-Quorum sensing、Antioxidation	(131)
*Lactobacillus plantarum* 2034	Ethyl Acetate Extract, (EAE)	*In Vivo*	Burn wound in diabetic rat model	Anti-MRSA biofilm effect and quorum sensing、Re-epithelialization、Increased levels of collagen、Angiogenesis	(132)
*Lactobacillus plantarum* MTCC 2621	CFS	*In Vivo*	Wound healing mice model	Anti-MRSA biofilm effect、Promotion of IL-6 and IL-10 secretion by THP-1 cells	(133)
Anti-oxidative stress effect					
*Lactobacillus kunkeei* NCHBL-003	CSK (Culture supernatant)	*In Vitro*	HaCaT cells、B16F10 cells	Reduction of ROS and MCIR、Increased level of MAPKs、Cell migration	(6, 126)
*Lactobacillus plantarum NIMBB003*	EPSs (Exopolysaccharides)	*In Vitro*	HDF cells	Antioxidation、Proliferation of HDF cells	(127)
*Limosilactobacillus reuteri* EIR/Spx-2	Lysate	*In Vitro*	L-929 cells、 RAW 264.7 cells	Reduction of ROS and H2O2、Increased expression levels of COLIAI、Antioxidation、Anti-inflammation	(128)
*Lactococcus chungangensis* CAU 1447	Heat-Killed	*In Vivo*	Wound healing in diabetic mice model	Reduction of MPO and ROS、Antioxidation、Regulation of inflammatory factors and growth factors、Macrophage migration	(129)
Bacillus subtilis					
*Bacillus subtilis* SPB1	Lipopeptide biosurfactant	*In Vivo*、*In Vitro*	Wound healing mice model	Antioxidation、Re-epithelialization	(134)
*Bacillus subtilis* D10	Proteases	*In Vivo*	Skin burn mice model	Accumulation of fibroblasts and keratinocytes、Increased levels of collagen、Re-epithelialization	(135)
*Bacillus subtilis sp. Natto* (*B.S. natto* ATCC 15245)	CS-NG (Lysate/Chitosan nanogel)	*In Vivo*	Wound healing mice model	Proteoglycan deposition、Angiogenesis、Anti-inflammation、Activation of growth factors:EGF PDGF FGF TGF-β	(133)
*Bacillus subtilis sp. natto* (*B.S. natto* ATCC 15245)	CFS	*In Vivo*	Wound healing mice model	Increased level of hydroxyproline and collagen、Re-epithelialization、Anti-inflammation	(134)
Yeast					
*Papiliotrema terrestris* PT22AV	EPSs (Exopolysaccharides)	*In Vivo*、*In Vitro*	Wound healing mice model/Human fibroblast、Macrophage	Antibacterial activity、Antioxidation、Facilitation of the recruitment of fibroblasts, keratinocytes, and macrophages、Transformation of M1 into phenotype M2 macrophages by ERK signaling pathway、Anti-inflammation	(135)
*Saccharomyces cerevisiae* CCMI 885	Fermentation supernatants	*In Vitro*	HUVEC cells	Antibacterial activity、Remodeling、Anti-inflammation	(136)
Enterococcus faecalis					
*Enterococcus faecalis strain* KH2	Heat-killed	*In Vivo*	Wound healing mice model	Anti-inflammation、Granulation tissue formation、Re-epithelialization	(137, 138)

### 5.3 Postbiotics of yeast metabolites promote wound repair

The use of yeast postbiotics can improve wound healing. In particular, the EPS from *Papiliotrema terrestris* PT22AV can transform M1 into phenotype M2 macrophages by stimulating the extracellular signal-regulated kinase (ERK) signaling route (137). Additionally, biotechnologically active peptides secreted from the fermentation adsorption supernatant of *Saccharomyces cerevisiae* CCMI 885 have antibacterial, antioxidant, ACE inhibitory and anti-inflammatory properties (138).

**Figure 2** illustrates the mechanisms by which postbiotics derived from lactic acid bacteria exert effects at different stages of wound healing, highlighting their biological activities in three key areas: regulating wound cell activity, inhibiting oxidative stress, and delivering antimicrobial and biofilm-disrupting effects. During wound initiation, postbiotics accelerate granulation tissue formation and re-epithelialization by enhancing fibroblast, vascular endothelial cell, and keratinocyte activity while promoting collagen and angiogenesis. During oxidative stress, postbiotics regulate inflammatory factor expression to facilitate wound healing by scavenging excess reactive oxygen species (ROS) or reducing myeloperoxidase (MPO) activity. During wound formation, the skin’s surface microbiota shifts toward pathogenic bacteria like Staphylococcus aureus and Pseudomonas aeruginosa. Postbiotics exert antibacterial effects by disrupting bacterial biofilms and interfering with quorum sensing, thereby promoting wound healing.

### 5.4 Postbiotics from *Enterococcus faecalis* promote wound repair

A Japanese research team found that heat-inactivated *Enterococcus faecalis* KH2 is recognized by Dectin-2 (a C-type lectin receptor, CLR), which activates the downstream signaling molecule CARD9, activates macrophages and dendritic cells, promotes the expression of inflammatory factors (such as TNF-α, IL-6) and chemokines (such as KC, MIP-2, MIP-1α/β), and promotes the migration of neutrophils and macrophages to the wound, thereby accelerating the transition from the inflammatory phase to the proliferative phase (139, 140).

### 5.5 Postbiotics from mixed microbial species promote wound repair

Kefir, a complex mixture of various bacteria, including lactic acid bacteria, acetic acid bacteria and yeast, aids wound healing by its metabolic products that act as postbiotics. In 2005, K. L. Rodrigues et al. showed an insoluble polysaccharide extracted from kefir-kefiran. While both kefir suspension and kefiran displayed antibacterial properties *in vitro*, kefiran emerged as the more effective agent. Translating this to an *in vivo* S. *aureus* wound infection model, kefiran effectively cleared the bacterial burden while simultaneously preserving the architectural integrity of the dermal matrix, underscoring its dual role in infection control and tissue protection (141).

### 5.6 Postbiotics from fermentation of herbal medicines promotes wound healing

Postbiotics derived from probiotics intrinsically exhibit a range of biological activities, including anti-inflammatory, antioxidant, and immunomodulatory effects, which facilitate their beneficial role in wound healing. Herbal Medicines contain hundreds of different components with diverse physiochemical properties based on metabonomic analysis (142). Postbiotics sourced from fermented herbal medicines extend these properties by integrating bioactive herbal constituents, resulting in a synergistic enhancement of therapeutic efficacy (143). Furthermore, in cases of more severe wounds involving structures such as hair follicles or even bone tissue, these postbiotics demonstrate a broader spectrum of biological functions, thereby significantly expanding the potential applications of postbiotics in advanced wound management and regenerative medicine.

A case in point is Chinese herbal medicines (CHM), as a key component of herbal medicine systems, has been used to treat diseases for thousands of years in China and other Asian countries, with its efficacy validated through long-term clinical practice (144). Therefore, fermented traditional Chinese medicine holds promising and broad prospects for future application.

#### 5.6.1 Anti-inflammatory and antioxidant activity

Probiotic-fermented herbal medicines combine microbial compounds with active herbs, and thus have synergistic effects and biotransformation benefits. **Table 3** summarizes the wound healing potential of fermented herbal medicine. For example, fermentation of Salvia Miltiorrhiza root with *Aspergillus oryzae* has shown that antioxidant and antibacterial active ingredients (fatty acids, phenolic compounds, and tanshinones) are increased, and fermentation of Matricaria chamomilla with *Lactobacillus plantarum* increases antioxidant and cytotoxic potential (145–147). Additionally, fermentation alters pharmacological effects, reduces herb toxicities, makes herbal palatability and improves overall quality of herbal formulations.

**Table 3:** Emerging promise: proposed mechanisms and applications for fermented herb postbiotics in wound healing.

Bacterial producer	Medicinal herb	Fermentation type	Experiment type	Cell/ Animal model	Mechanism	References
*Aspergillus oryzae* KCTC 6983	Roots of Salvia miltiorrhiza	Solid-state fermentation	*In Vitro*		Antioxidation、Antibacterial activity(S. aureus).	(145)
*Lactobacillus plantarum* KCCM 11613P	Matricaria chamomilla	Liqued-state fermentation	*In Vitro*		Antioxidation	(146, 147)
*Bacillus subtilis* N2	Pine needle	Liqued-state fermentation	*In Vitro*	RAW 264.7 cells	Antioxidation、Anti-inflammation	(148)
Yeast	Safflower	Solid-state fermentation	*In Vivo*、*In Vitro*	RAW264.7 cells、 HaCaT cells/Zebrafish inflammation model	Antioxidation、Anti-inflammation	(149)
*Bacillus licheniformis*、*Lactobacillus plantarum*、*Saccharomyces cerevisiae*、*Eurotium cristatum*	White Ginseng	Solid-state fermentation	*In Vitro*		Antioxidation	(150)
*Lactobacillus casei* GDMCC1.159、*Lactobacillus rhamnosus* ATCC7469、*Lactobacillus plantarum* GDMCC1.648	Ginseng	Solid-state fermentation	*In Vitro*	HaCaT cells、BJ cells	Antioxidation	(151)
*Isaria cicadae Miquel* CGMCC 13684	Rice	Solid-state fermentation	*In Vivo*	Wound healing mice model	Reduction in inflammatory cells、Re-epithelialization、Promotion scarless healing、Hair follicle regeneration	(19)
*Lactobacillus rhamnosus*	ChiBai	Liqued-state fermentation	*In Vivo*、*In Vitro*	B16F0 cells/ UVB-treated mice model	Anti-melanogenic by CREB/MITF/ tyrosinase signaling pathway	(153)
*Aspergillus oryzae* BCRC 32288	Asparagus cochinchinensis	Liqued-state fermentation	*In Vitro*	CCD-966SK、A375.S2、HaCaT cells	Antioxidation、Anti-tyrosinase activity	(154)
*Mucor circinelloides* T2-12	Stemonae Radix	Solid-state fermentation	*In Vitro*		Antioxidation、Anti-tyrosinase activity	(155)
*Lactiplantibacillus plantarum* CCFM1348	Cacumen platycladi	Solid-state fermentation	*In Vivo*、*In Vitro*	HDPC cells/ Hair Loss Mice Model	Promotion of hair growth	(103)
*Bacillus subtilis* MV1、*Bacillus velezensis* MV2、*Bacillus subtilis* 168	Malva verticillata Leaves、Astragalus	Liqued-state fermentation	*In Vitro*	C3H10T1/2 cells、RAW264.7 cells	Antioxidation、Osteogenic effect	(156)

Fermented herbal medicines can treat skin diseases through multiple pathways, including anti-inflammation, anti-oxidation, promotion of collagen production and cell activities. For example, the ethyl acetate extract (Ethyl Acetate Extract, EAE-FPN) obtained from the fermentation of pine needles with *Taiwanofungus camphoratus* (TC) contains higher levels of phenols and flavonoids than the water extract. It can effectively eliminate ROS, inhibit the NF-κB signaling pathway, and significantly reduce the levels of key inflammatory factors such as NO, PGE2, TNF-α, IL-1β/6 (148). Herbal medicines rich in flavonoids and polysaccharides, such as safflower, have their fermented products widely used in cosmetics due to their antioxidant and anti - inflammatory activities (149).

Moreover, the fermentation strategy can significantly affect the active components and efficacy of herbal medicines. Studies have shown that after solid - state fermentation of white ginseng using different strains, including *Bacillus coronus*, *Bacillus licheniformis*, *Lactobacillus plantarum*, and *Saccharomyces cerevisiae*, the total flavonoid content, ferric reducing antioxidant power (FRAP), DPPH radical scavenging activity, and ABTS radical scavenging activity are all significantly improved (150). Notably, different strains have different effects on the components: fermentation with *Bacillus coronus* and *Bacillus licheniformis* increases the total phenol content, while fermentation with *Lactobacillus plantarum*, *Saccharomyces cerevisiae*, and mixed strains decreases it. Metabolomic analysis further reveals significant differences in metabolites related to antioxidation, such as carboxylic acids, flavonoids, polyphenols, and coumarins.

Similarly, the combined fermentation of ginseng stem and leaf extract with five strains of lactic acid bacteria optimizes multiple antioxidant indexes. Experiments on human skin cell models (such as HaCaT and fibroblast BJ cells) have confirmed that it can effectively scavenge free radicals and prevent and repair oxidative damage (151). These findings indicate that probiotic fermentation is an effective strategy to enhance the biological activity of herbs.

#### 5.6.2 Regulating cell activity to promote wound healing

While research on the role of fermented herbal medicines in promoting wound healing remains in its nascent stages, there is emerging evidence of their potential to enhance active components and modulate multicellular pathways involved in the healing process.

Promoting Repair and Regeneration

The fermented rice extract from *Isaria cicadae Miquel* CGMCC 13684 contains high amounts of amino acids and nucleosides. The amino acids stimulate collagen production, while nucleosides are used for re-epithelialization. Network Pharmacology, supported by animal studies, predicts that this extract can promote full-thickness skin defect models in ICR mice through GPCR, EGFR and Hippo signaling, as well as hair follicle regeneration (19).

Inhibition of Pigmentation

Wound healing is often associated with hyperpigmentation. In C.C. Ho et al., a seven-herb formula containing ChiBai fermented with *Lactobacillus rhamnosus* inhibited melanin production by targeting the CREB/MITF/tyrosinase signaling pathways. This inhibition inhibits melanin production caused by α-MSH and UVB exposure (152). The extracts of Mucor T2–12 fermented Stemonae Radix and *Aspergillus oryzae* fermented Asparagus cochinchinensis showed similar anti-tyrosinase activity and antioxidant activity (153, 154).

Stimulating Hair Growth

Hair follicles, which form the skin appendages, play a vital role in wound healing because they are regenerable. Recent studies have shown that fermentation of Cacumen platycladi leaves by *Lactobacillus plantarum* stimulates dermal papilla cells by raising β-catenin, which is a component of Wnt/β-catenin signaling pathway, and also enhances the expression of vascular endothelial growth factor and modulates BAX/Bcl-2 to reduce apoptosis, which leads to hair growth in murine models (103).

Enhanced osteogenic activity

Tong et al. highlighted the biological significance of mixed herbal fermentation in the context of bone trauma, extending beyond the scope of single fermentation processes. Fermentation of Astragalus membranaceus and Malva verticillata with *Bacillus subtilis* enhances both its antioxidant activity and osteoblast differentiation activity. Mechanistic studies show that fermented product stimulates the differentiation of C3H10T1/2 cells into osteoblasts via TGF-β signaling and p38 phosphorylation (155).

## 6 Summary and outlook

Challenges:

Unclear preparation process and dose - effect relationship: The interactive effects of fermentation conditions (time, temperature, pH, and inoculum size) result in unstable bioactive components and an unclear dose - effect relationship (156). Therefore, systematic research is required to optimize the process.

Lack of supervision and quality standards: Currently, postbiotics are not regarded as drugs by the U.S. Food and Drug Administration (FDA), the European Medicines Agency (EMA), or other institutions (157, 158). There is a lack of unified supervision and quality standards for fermented herbal medicine postbiotics (18). This causes uneven product quality, and it is urgent to establish a quality control marker system based on key active components. Although widely recognized, some studies have found that metabolites of certain strains, such as the secondary metabolite colicin of Escherichia coli Nissle 1917 (EcN), may have potential toxic effects (157). Currently, there is a lack of large-scale clinical trials to verify their safety and effectiveness, making it difficult to meet the high-standard clinical requirements.

Scarcity of Clinical Microbiome Biomarkers: Although dysbiosis is implicated in numerous cutaneous and systemic diseases, the clinical utility of postbiotics remains constrained by the paucity of specific diagnostic biomarkers, precluding precise identification of intervention windows and beneficiary populations. Accordingly, we aim to pivot towards clinical translation by elucidating the molecular determinants of dysbiosis to identify actionable targets, thereby advancing postbiotic therapy from empirical application to multi-target, standardized precision medicine.

Opportunity:

Postbiotics contain a variety of active ingredients, which can intervene in the complex healing process through multiple mechanisms such as anti-inflammation, anti-bacteria, immune regulation, and promotion of cell proliferation and migration. This multi-mechanism approach is more advantageous than single-target therapies.

Standardization and industrialization potential: With the in-depth research, the production of postbiotics can be standardized in GMP facilities. By establishing industry, national, and international standards and improving the safety and functionality evaluation systems, the stability of product quality can be ensured, laying a foundation for industrialization.

Technological innovation and product diversification: In the future, the functions of postbiotics can be enriched by exploring new medicinal plant raw materials and probiotic strains; the yield of active ingredients can be increased by optimizing fermentation processes; and new products with special flavors and nutritional and health benefits can be developed to meet diverse needs, injecting new vitality into skin health and related fields.

Potential of Postbiotics in Managing Complex Dermatoses: The polypharmacological nature of postbiotic formulations provides a robust theoretical and practical foundation for expanding their therapeutic scope. By enabling niche reconstruction, multi-target immunomodulation, barrier functional restoration, and systemic regulation via the gut–skin axis, postbiotics are uniquely positioned to address the heterogeneity of complex skin diseases. Future endeavors should aim to extend this precision microbiome therapy beyond dermatological indications to systemic disorders, thereby offering novel paradigms for clinical intervention.
